# The role of tumor necrosis factor alpha − 308A > G polymorphism on the clinical states of SARS-CoV-2 infection

**DOI:** 10.1186/s43042-022-00274-0

**Published:** 2022-03-07

**Authors:** Francisco Sotomayor-Lugo, Claudia Alemañy-Díaz Perera, Hilda Roblejo-Balbuena, Yaíma Zúñiga-Rosales, Giselle Monzón-Benítez, Beatriz Suárez-Besil, María de los Ángeles González-Torres, Bárbara Torres-Rives, Yudelmis Álvarez-Gavilán, Maidalys Bravo-Ramírez, Nayade Pereira-Roche, Yudelkis Benítez-Cordero, Luis Carlos Silva-Ayçaguer, Beatriz Marcheco-Teruel

**Affiliations:** 1 National Center of Medical Genetics, Havana, Cuba; 2 Medical University of Havana, Havana, Cuba; 3 National School of Public Health, Havana, Cuba

**Keywords:** Coronavirus infections, COVID-19, Severe acute respiratory syndrome coronavirus 2, SARS-CoV-2, Tumor necrosis factor alpha

## Abstract

**Background:**

Tumor necrosis factor-alpha (TNFɑ) is a cytokine that manages the host defense mechanism, which may play a role in the pathogenesis of COVID-19 patients. Several single-nucleotide polymorphisms, described in the promoter region of the TNFα gene, have a significant role on its transcriptional activity. These include the − 308A > G polymorphism which increases the TNFα levels with the expression of the A allele. The aim of this study was to explore whether the TNFα.− 308A > G polymorphism affects the clinical state of COVID-19 patients. The study included a total of 1028 individuals infected with severe acute respiratory syndrome coronavirus 2 (SARS-CoV-2), which were distributed in 3 groups: asymptomatic, mild symptomatic and severe symptomatic patients. The amplification-refractory mutation system was used to determine the genotype of the TNFα.− 308A > G polymorphism.

**Results:**

Results show a higher tendency of being asymptomatic in individuals carrying the GG genotype (336 of 411; OR 1.24, 95% CI 0.91–1.70). The development of a severe form of SARS-CoV-2 infection was not found in subjects with the A allele compared to those with the G allele (OR 0.96, 95% CI 0.51–1.79), except in the eastern region of the country where the risk increased (OR 4.41, 95% CI 1.14–17.05). However, the subjects carrying the A allele had a higher chance of developing symptoms (OR 1.24, 95% CI 0.91–1.70) compared to those with the G allele.

**Conclusion:**

The TNFα.− 308A allele has an influence on developing symptoms of COVID-19 in Cuban patients, and that it particularly increases the risk of presenting severe forms of the disease in the eastern region of the country.

## Background

Severe acute respiratory syndrome coronavirus 2 (SARS-CoV-2) has caused a pandemic with devastating consequences for public health and health systems worldwide. Factors triggering the severe illness in individuals infected with SARS-CoV-2 have not been completely identified. The development of severe disease forms of the illness does not seem to be related to only one factor [1, 2]. An excessive inflammatory response to SARS-CoV-2 is thought to be a major cause of disease severity in patients with coronavirus disease 2019 (COVID-19) [3, 4]. It is associated, among other factors, to high levels of circulating cytokines including the tumor necrosis factor-alpha (TNFα) [5], which could express an unbalanced and harmful response.

TNFα has a potent pro-inflammatory action, exerting pleiotropic effects on various cell types and it plays a dual role, since it acts as an agent for both innate immunity and inflammatory pathology [6, 7]. TNFα regulation, carried out in the promoter region of the gene, constitutes a considerable challenge for the molecular machinery. Several single-nucleotide polymorphisms (SNPs), described in the promoter region of the TNFα gene [8], have a significant role on its transcriptional activity. These include the − 308A > G (rs1800629) polymorphism which increases the TNFα levels with the expression of the A allele [9]. However, the overexpression of this cytokine can produce an unfavorable course of the diseases, including COVID-19.

The purpose of this paper is to learn if the TNFα − 308A > G polymorphism can have a major influence on the clinical state of patients with SARS-CoV-2 infection. This infection varies from individual to individual, some can be asymptomatic carriers or they may present mild symptoms, while others can suffer lethal forms that, even with timely medical interventions, can rapidly evolve into complications. This possible relationship could be useful in establishing clinical prognosis and personalized treatments for patients with SARS-CoV-2 infections, and it can help outline population-based prevention strategies.

## Methods

### Subjects

The study included 1028 Cuban patients infected with SARS-CoV-2. Six hundred seventeen patients developing the symptomatic form of COVID-19 were included; they were randomly selected from provincial records reported to the Cuban Ministry of Public Health. The patients were divided into two groups, 551 with mild symptoms who were admitted to the ordinary ward and 66 requiring intermediate or intensive care due to complications of the disease. A comparison group of 411 asymptomatic subjects with documented SARS-CoV-2 infection, also randomly selected, was included in the study. All individuals were infected with SARS-CoV-2 that was confirmed by reverse transcription-polymerase chain reaction (RT-PCR), with a clinical window of 15–90 days since the diagnosis. Children under 1 year of age and deceased patients were excluded.

### Ethical commitment

The Scientific Council and Medical Ethics and Scientific Research Committee of the National Center of Medical Genetics received the study protocol, and it was approved according to guidelines and standards. All patients gave their written informed consent and the analyses were carried out on anonymous data, as required.

### Molecular characterization

Automatic DNA extraction using QIA symphony DNA Mini (Qiagen) was performed, pursuant to the standard operating procedure for DNA Blood 200 extraction according to the manufacturer's instructions. The DNA extracted was quantified and frozen at − 20 °C for preservation. Primers were designed according to Sotomayor et al. [10] Fragments of 184 base pairs was amplified with forward primers F1: 5′-ATAGGTTTTGAGGGGCATGA-3′, F2: 5′-ATAGGTTTTGAGGGGCATGG-3′ and reverse primer R: 5′-TCTCGGTTTCTTCTCCATCG-3′. The amplification-refractory mutation system (ARMS) was standardized. One region of the *FGFR3* gene was used as the internal amplification control, with fragments of 164 base pairs led by primer sequences 5′-GGAGATCTTGTGCACGGTGG-3′ and 5′-GGAGATCTTGTGCACGGTGG-3′. The standard 25 μl ARMS containing 2.5 μl QIAGEN 10 × PCR Buffer, 1.875 μl (2.0 μM) Deoxynucleotide Mix, 1.325 μl (10.0 μM) forward and reverse primers, 0.5 μl (8.0 μM) primers of internal amplification control, 0.2 μl Hot Star Taq DNA Polymerase (QIAGEN), 2 μl DNA template. Amplification was carried out in a thermal cycler (MJ Research Inc., MA) with cycle parameters of 4 min at 94 °C (initial denaturation), 29 rounds of 45 s at 94 °C (denaturation), 45 s at 55 °C (hybridization), 45 s at 72 °C (extension) and a final extension for 5 min at 72 °C. The reaction products were identified in 2% agarose gel electrophoresis.

### Population genetics analysis

The allele and genotype frequencies of the TNF − 308A > G polymorphism were calculated using GENEPOP 4.4 for Windows/Linux/MacOsX (2015). The data were checked for the Hardy–Weinberg equilibrium by calculating the expected frequencies of each genotype and comparing them to the observed values. A Chi-square analysis was used to test the goodness of fit between the observed and expected values. All frequency data for genotypes and alleles were compared to those of the population reference frequencies of Cuba and its provinces using z test with the Yates correction. The reference sample data included 984 individuals genotyped for the site − 308 of the TNFα gene that were part of a nationwide study to explore the history of admixture and the genetic basis of pigmentation present in today’s Cuban population, which was carried out by the National Medical Genetics Center [11].

### Statistical analysis

The statistical analysis was carried out using the Statistical Package for Social Sciences (SPSS) version 21.0 and GraphPad Prism version 8.0.1. Categorical variables were computed as proportions with 95% confidence intervals (CI). Continuous variables were described as means with 95% CI, or medians and interquartile ranges (IQR). Chi-square was used to analyze differences in the distribution of alleles and genotypes between subgroups of clinical forms of SARS-CoV-2 infection. Univariate analysis and multivariate logistic regression were used to calculate the odds ratios (ORs) and the 95% CI to study the relationship between the TNFα − 308.A allele and forms of the clinical presentation of COVID-19.

## Results

### Demographic characteristics

Table 1 shows the baseline characteristics of patient groups with SARS-CoV-2 infection. The median (IQR) age of people with SARS-CoV-2 infection was 46 (29.0–57.0) years old. We observed that in subgroups of individuals 60 years old or older, as age increased, patients were more likely to suffer from a severe form of the disease. The results of gender showed that the male/female ratio was 1:1.18. As to skin pigmentation, the number of white skin color patients was 649 (63.1%), while the number of mestizo individuals was twice the number of black skin color subjects, 241 (23.4%) and 131 (12.7%), respectively. The most frequent comorbidity of all groups at the time of hospitalization was hypertension (HT), which affected 35.5% (365) of all patients. The most common symptoms at the time of hospitalization were fever (301 [29.3%]), cough (247 [24.0%]), loss of smell (187 [18.2%]) and loss of taste (187 [18.2%]), followed by shortness of breath (163 [15.9%]), fatigue (156 [15.2%]), sore throat (147 [14.3%]), diarrhea (140 [13.6%]), headache (59 [5.7%]) neurological symptoms (43 [4.2%]) and breathing pain (38 [3.7%]).

**Table 1:** Baseline characteristics of groups of patients with SARS-CoV-2 infection

Characteristics	Patients no. (%)		
	Asymptomatic (*n* = 411)	Mild symptomatic (*n* = 551)	Severe symptomatic (*n* = 66)
Age			
< 20	60 (14.6)	56 (10.2)	0 (0,0)
20–29	66 (16.1)	90 (16.3)	5 (7.6)
30–39	73 (17.8)	79 (14.3)	2 (3.0)
40–49	57 (13.9)	92 (16.7)	9 (13.6)
50–59	92 (22.4)	115 (20.9)	16 (24.2)
≥ 60	63 (15.3)	119 (21,6)	34 (51.5)
Median (IQR)	41 (26.0–54.0)	46 (29.0–58.0)	60 (50.5–71.5)
Sex			
Male	193 (47.0)	251 (45.6)	28 (42.4)
Female	218 (53.0)	300 (54.4)	38 (57.6)
Skin color^a^			
White	256 (62.3)	347 (63.0)	46 (69.7)
Mestizo	103 (25.1)	125 (22.7)	13 (19.7)
Black	51 (12.4)	73 (13.2)	7 (10.6)
Comorbidity			
Hypertension	108 (26.3)	217 (39.4)	40 (60.6)
Asthma	51 (12.4)	93 (16.9)	10 (15.2)
Diabetes	22 (5.4)	61 (11.1)	15 (22.7)
Obesity	22 (5.4)	34 (6.2)	6 (9.1)
Cardiovascular disease (not including HT)	18 (4.4)	39 (7.1)	13 (19.7)
Hypercholesterolemia	12 (2.9)	19 (3.4)	3 (4.5)
Cancer	7 (1.7)	14 (2.5)	3 (4.5)
COPD	6 (1.5)	15 (2.7)	6 (9.1)
Bronchiectasis	1 (0.2)	3 (0.5)	2 (3.0)
Sleep apnea	0 (0.0)	1 (0.2)	1 (1.5)

*IQR* interquartile range, *HT* hypertension, *COPD* chronic obstructive pulmonary disease

^a^Missing value: 0.7%

### TNFα − 308A > G polymorphism

Genotype and allele frequencies of TNFα − 308A > G polymorphism in the reference population are shown in Table 2. Hardy–Weinberg analysis of the data indicated it was in equilibrium (*p* > 0.05). The genotype frequencies in the population studied were adjusted to those of the reference population, except for Holguín province. Compared to local reference frequencies, the population studied in Holguín had an increased frequency of the TNFα− 308.GG genotype and a decreased frequency of the TNFα − 308.AG genotype.

**Table 2:** Genotype and allele frequencies of TNFα − 308A > G polymorphism by province in Cuba

Location		Reference group frequencies	Study group frequencies	Location		Reference group frequencies	Study group frequencies	Location		Reference group frequencies	Study group frequencies
Cuba		*n* = 948	*n* = 1028	Matanzas		*n* = 68	*n* = 116	Las Tunas		*n* = 43	*n* = 13
Genotype	GG	76.7	79.7	Genotype	GG	73.5	82.7	Genotype	GG	81.4	84.6
	AG	21.7	18.4		AG	25.0	14.7		AG	18.6	15.4
	AA	1.6	1.9		AA	1.5	2.6		AA	0	0.0
Allele	G	0.88	0.89	Allele	G	0.86	0.90	Allele	G	0.91	0.92
	A	0.12	0.11		A	0.14	0.10		A	0.09	0.08
Isle of Youth		*n* = 2	*n* = 34	Villa Clara		*n* = 101	*n* = 133	Holguín		*n* = 116	*n* = 54
Genotype	GG	50.0	76.5	Genotype	GG	79.2	78.2	Genotype	GG	73.3	88.9^a^
	AG	50.0	23.5		AG	19.8	20.3		AG	25.0	11.1^a^
	AA	0.0	0.0		AA	1.0	1.5		AA	1.7	0.0
Allele	G	0.75	0.88	Allele	G	0.89	0.88	Allele	G	0.86	0.94
	A	0.25	0.12		A	0.11	0.12		A	0.14	0.06
Pinar del Río		*n* = 79	*n* = 36	Cienfuegos		*n* = 29	*n* = 18	Granma		*n* = 88	*n* = 8
Genotype	GG	75.9	88.9	Genotype	GG	72.4	77.8	Genotype	GG	84.1	100.0
	AG	19.0	11.1		AG	27.6	22.2		AG	14.8	0.0
	AA	5.1	0.0		AA	0	0.0		AA	1.1	0.0
Allele	G	0.85	0.94	Allele	G	0.86	0.89	Allele	G	0.91	1.00
	A	0.15	0.06		A	0.14	0.11		A	0.09	0.00
Artemisa		*n* = 29	*n* = 22	Sancti Spíritus		*n* = 58	*n* = 54	Santiago de Cuba		*n* = 98	*n* = 32
Genotype	GG	72.4	72.7	Genotype	GG	70.7	74.1	Genotype	GG	74.5	84.4
	AG	27.6	27.3		AG	29.3	24.1		AG	23.5	12.5
	AA	0.0	0.0		AA	0	1.8		AA	2.0	3.1
Allele	G	0.86	0.86	Allele	G	0.85	0.86	Allele	G	0.86	0.91
	A	0.14	0.14		A	0.15	0.14		A	0.14	0.09
Mayabeque		*n* = 28	*n* = 27	Ciego de Ávila		*n* = 34	*n* = 74	Guantánamo		*n* = 57	*n* = 13
Genotype	GG	85.7	74.1	Genotype	GG	70.6	71.6	Genotype	GG	78.9	84.6
	AG	14.3	25.9		AG	29.4	24.3		AG	21.1	7.7
	AA	0	0.0		AA	0	4.05		AA	0	7.7
Allele	G	0.93	0.87	Allele	G	0.85	0.84	Allele	G	0.89	0.88
	A	0.07	0.13		A	0.15	0.16		A	0.11	0.12
Havana		*n* = 74	*n* = 363	Camagüey		*n* = 44	*n* = 31				
Genotype	GG	73.0	79.6	Genotype	GG	88.6	77.4				
	AG	23.0	18.2		AG	9.1	19.4				
	AA	4.0	2.2		AA	2.3	3.2				
Allele	G	0.84	0.89	Allele	G	0.93	0.87				
	A	0.16	0.11		A	0.07	0.13				

Frequencies of TNFa  − 308  genotypes are presented in percentages, and allele frequencies are showed in proportions

*n* number of subjects

^a^Differences by the z test

The analyses of genotype and allele frequencies according clinical severity showed that the AA homozygote frequency of the TNFα − 308A > G polymorphism was of 1.2% and 0.8% in symptomatic subjects and the asymptomatic comparison group, respectively. Additionally, the frequency of the distribution of the AG genotype for symptomatic and asymptomatic patients was 11.9% and 6.5%, respectively. However, the genotype frequency of the individuals carrying the A allele was 7.3% in asymptomatic patients, 11.8% in mild symptomatic patients and 1.3% in severe symptomatic patients (Table 3). Concerning the degree of disease severity, we found a higher tendency of developing mild symptoms in individuals carrying the AG genotype (OR 1.26, 95% CI 0.91–1.73). However, no significant differences were found for the AG genotype in patients with a mild disease compared to those with severe COVID-19 (110 out of 551 patients versus 12 out of 66 patients; OR 0.89, 95% CI 0.46–1.72). Likewise, the frequency of the GG genotype in symptomatic (mild and severe) and asymptomatic patients was similar. However, we found that carriers of the GG genotype had a higher tendency to have no symptoms (336 of 411; OR 1.24, 95% CI 0.91–1.70).

**Table 3:** Genotype frequencies and *odds* ratios in degree of severity and clinical forms of COVID-19 according to TNFα − 308A > G polymorphism genotypes

Characteristics	Genotypes					
	GG (%)	OR (95% CI)	AG (%)	OR (95% CI)	AA (%)	OR (95% CI)
Severity of symptomatic forms of COVID-19						
MS (*n* = 551)	430 (78.0)	0.81 (0.59–1.09)	110 (20.0)	1.26 (0.91–1.73)	11 (2.0)	1.06 (0.44–2.58)
SS (*n* = 66)	53 (80.3)	1.04 (0.56–1.95)	12 (18.2)	0.99 (0.52–1.88)	1 (1.5)	0.76 (0.10–5.79)
Clinical forms						
MS&SS (617)	483 (78.3)	0.81 (0.59–1.10)	122 (19.8)	1.27 (0.91–1.76)	12 (1.9)	0.99 (0.41–2.47)
AS (*n* = 411)	336 (81.8)^a^	1.24 (0.91–1.70)	67 (16.3)^a^	0.79 (0.57–1.10)	8 (1.9)	1.00 (0.41–2.47)

Frequencies of TNFa − 308 genotypes are presented in absolute values and percentages

*AS* asymptomatic, *MS* mild symptomatic, *SS* severe symptomatic

^a^Differences by the z test when compared with the Cuban reference population

### Influence of TNFα − 308 polymorphism genotypes on the number of days from the positive test to SARS-CoV-2 by RT-PCR until hospital discharge

**Fig. 1:**
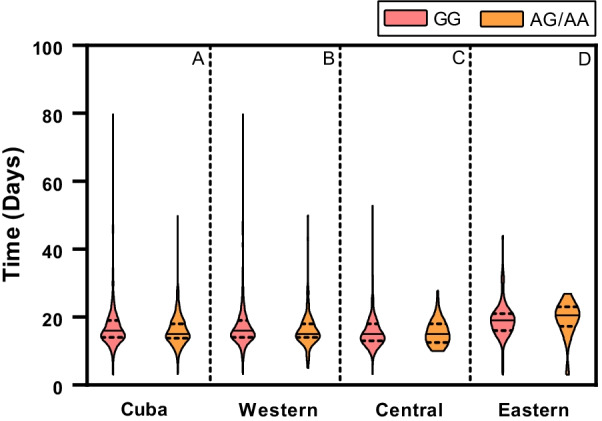
Number of days from the positive RT-PCR test for SARS CoV-2 until hospital discharge according to TNFα− 308A > G polymorphism genotypes. Violin plots showing hospital stay as of the time of the positive RT-PCR test in Cuba (*n* = 1028, included = 955: GG = 761, AG/AA = 194; missing value = 7.1%) (**A**), Western region (*n* = 598, included = 541: GG = 434, AG/AA = 107; missing value = 9.5%) (**B**), Central region (*n* = 310, included = 306: GG = 233, AG/AA = 73; missing value = 1.3%) (**C**) and the Eastern region (*n* = 120, included = 108: GG = 94, AG/AA = 14; missing value = 10.0%) (**D**). Time differences according to genotype frequency by region were not observed

The effect of TNFα − 308 polymorphism on the period from the day of the positive test to SARS-CoV-2 by RT-PCR until hospital discharge, was studied on all infected individuals, according to their genotype and by the region of the country. The number of infected individuals with the GG genotype always had the highest frequency, followed by AG and AA individuals. These results are similar to the distribution of the reference genotypes in Cuba. No differences were found in the number of days according to genotype frequency (Fig. 1A). The median of the days of hospitalization according genotype was 16 days for GG (range, 3–80 days; 25% and 75% IQR: 14–19 days), 15 days for AG (range, 3–50 days; 25% and 75% IQR: 13–19 days) and 15 days for AA (range, 6–21 days; 25% and 75% IQR: 14–17 days).

For a better understanding of the effect of the A allele on the number of days from the SARS-CoV-2 positive test until hospital discharge, the median of the number of days was analyzed according to the presence or absence of the A allele in individuals stratified by region. In the western area, the median of the number of days for GG individuals was 16 days (range, 3–80 days; 25% and 75% IQR: 14–19 days) and in individuals with the AG/AA genotypes, it was 15 days (range, 5–50 days; 25% and 75% IQR: 14–18 days) (Fig. 1B). The median of the number of days in the central region was 15 days for GG individuals (range, 3–53 days; 25% and 75% IQR: 13–18 days) and 15 days to AG/AA (range, 10–28 days; 25% and 75% IQR: 13–18 days) (Fig. 1C). In the eastern region, the median of the number of days was 19 days for subjects with the GG genotype (range, 3–44 days; 25% and 75% IQR: 16–21 days) and 21 days for those with the AG/AA genotype (range, 3–27 days; 25% and 75% IQR: 17–23 days) (Fig. 1D).

### Association between the TNFα − 308.A allele and the clinical states of SARS-CoV-2 infection

As shown in Table 4, Cuban patients infected with the SARS-CoV-2 virus had, although not dramatically, a higher chance of presenting COVID-19 symptoms when they were carrying the − 308.A allele of the TNFα gene. There is no evidence of an increased risk of developing severe forms of the disease in carriers of the − 308.A allele. Differences were, however, observed in the eastern region of the country, i.e. there was an increased risk of four more units in the *odds* ratio for developing severe forms of COVID-19 in individuals carrying the A allele. The results of the logistic regression for the risk adjusted by comorbidity (hypertension, diabetes and obesity) did not show a different behavior.

**Table 4:** Crude and adjusted *odds* ratios for different levels of severity of SARS-CoV-2 infection according to the presence of the TNFα − 308.A allele by region

Characteristics	Symptomatic versus asymptomatic cases		Severe symptomatic versus non-severe symptomatic cases	
	Crude OR (95% CI)	Adjusted OR (95% CI)^a^	Crude OR (95% CI)	Adjusted OR (95% CI)^a^
Cuba	1.24 (0.91–1.70)	1.22 (0.89–1.68)	0.96 (0.51–1.79)	0.93 (0.49–1.75)
Eastern region	4.00 (0.86–18.66)	4.05 (0.84–19.43)	4.41 (1.14–17.05)	4.62 (1.11–19.26)
Central region	1.15 (0.68–1.93)	1.14 (0.67–1.95)	0.83 (0.27–2.57)	0.75 (0.23–2.42)
Western region	1.29 (0.84–1.99)	1.27 (0.82–1.98)	0.65 (0.25–1.73)	0.61 (0.23–1.64)

^a^OR was adjusted for comorbidities such as hypertension, diabetes and obesity

## Discussion

Several studies have expressed that the hyper-inflammatory response induced by SARS-CoV-2 is a major cause of disease severity and death [6, 12, 13]. Levels of interleukin TNF-α were reported in patients with severe COVID-19, although other reports [14, 15] suggest that other cytokines are involved in the pathogenesis of the illness. In order to explore the factors in the host, with the strongest effect on the clinical progression of COVID-19, we studied the − 308A > G polymorphism of TNFα gene located at the promoter region, which has been ascribed to the polymorphisms within the regulatory regions [16]. It results in two allelic forms, one in which guanine defines the common allele (TNFα.G) and the other in which guanine is replaced by adenosine, which behaves as the rarer allele (TNFα.A) at position − 308. The presence of the rarer allele was found to correlate with enhanced spontaneous or stimulated TNF-α production [17].

When comparing genotype and allele frequencies of TNFα− 308A > G polymorphism in individuals having SARS-CoV-2 infection, with those of the reference population, no relevant differences were found. This led us to infer that persons carrying the A allele are not more susceptible to SARS-CoV-2 infection. This contrasts with the report by Ahmed Saleh et al., who found that the A allele is expressed more frequently in patients than in the controls, and that individuals with AA and AG genotypes are more susceptible to COVID-19 [18]. We acknowledge that a limitation in our study is the fact that we did not control the allele and genotype frequencies according to ethnic background.

However, when the reference frequencies in Holguín province were compared to the frequencies of our study in that province, the distribution of genotypes differed, mainly due to an increased frequency of TNFα.G allele homozygotes. The low frequency of the TNFα.AA genotype in this population may be due to natural selection favoring individuals with a low production potential instead of those with a high secretion of TNFα. An important implication of the Hardy–Weinberg Law is that, for a rare allele, the frequency of heterozygotes is much higher than the unusual homozygote. This argument proves that it is difficult to eliminate recessive deleterious alleles from the population, since most of them are in a heterozygous state.

To better understand the differences in the clinical forms of SARS-CoV-2 infection, we compared the TNFα − 308A > G polymorphism genotypes according to disease severity. Our results suggest that the TNFα.AG genotype seems to be related to symptomatic forms of the disease, which could be explained considering that TNFα mediates in many symptoms such as fever [19], dry cough [20], muscle weakness [21, 22] and multiple organ disfunction [23], all of which are present in COVID-19 [24]. However, we found that the TNF.AA genotype may not be a relevant factor in more severe forms, although the number of homozygotes for the A allele was very low in individuals with severe forms of the disease. In addition, we found a higher tendency of being asymptomatic in the G allele homozygous individuals infected with SARS-CoV-2. When the genotype frequencies of the TNFα − 308A > G polymorphism in asymptomatic, mild symptomatic and severe symptomatic groups were compared to those of the reference population, random distribution was not found in the asymptomatic cluster, where the frequency of the TNFα − 308.AG genotype was lower and there was an increase of the TNFα − 308.GG genotype. This led us to infer that individuals with the GG genotype could be predisposed to develop asymptomatic forms of SARS-CoV-2 infection.

Reports show that patients with SARS-CoV-2 infection can develop a symptomatic or asymptomatic form of the disease, but most COVID-19 cases are symptomatic with a moderate fatality rate [25]. A large proportion of COVID-19 patients present common symptoms including fever, cough, sore throat, nasal congestion, weakness, fatigue or myalgia, dizziness, shortness of breath, muscle pain, arthralgia, chest tightness, overproduction of mucus with expectoration, hemoptysis and dyspnea [24] Our study agrees with others concluding that fever and cough are the most prevalent symptoms of the disease, in that order, although we did not find similar results regarding fatigue as the third most frequent symptom [26, 27]. Smell and taste loss were also frequent symptoms in our population, which agrees with others reporting these as the fourth most common symptom of SARS-Cov-2 infection [28]. The approximate proportion of severe versus mild symptomatic patients in our study was estimated as 1:8, it differs from that reported by other researchers with estimates of 1:4 [29].

COVID-19 has an average duration of approximately 6 weeks [24]. However, we observed a high variability in our COVID-19 patients. Recovery time was highly variable, with an average duration of 17.1 ± 6.8 days, which differs from other studies reporting a duration of more than 20 days [30, 31]. This finding may be related to the early start of therapeutic protocols in Cuba, as a result of an efficient active tracing of cases in the communities for the epidemiological control of active cases and their contacts. However, it is noteworthy that in the eastern region of the country, the median of the number of days from diagnosis to hospital discharge (after a negative test by RT-PCR) was higher than in the other regions. The presence of the A allele increases the number of days hospitalized in this region, but because of sample size, new studies with a larger number of individuals are recommended. This result is similar to that reported by Angioni et al. [14] who relates the high levels of TNFa with hospitalization time.

The analysis of the risk of a severe COVID-19 clinical form, according to region, with the presence of the A allele, showed a relationship between the polymorphism and disease severity only for the eastern region of Cuba, which is similar to what was reported in Mansoura (Egypt) where it was associated with a more severe disease [18]. Our results suggest that the presence of the A allele in the eastern Cuban population could be associated to a more symptomatic form of the disease and this may be related to variations in TNF-ɑ levels in the serum as a key mediator of the inflammatory response. However, the risk of developing COVID-19 symptoms had a tendency to increase in all regions, although not dramatically, in individuals carrying the − 308.A allele, even when analyzing this polymorphism accompanied by comorbidities [32] associated to the severity of the disease. Although plasma levels of TNF-α may be submitted to a multifactorial regulatory process, the local TNF-α concentration involved in the pathogenesis of COVID-19 [15] may be under a major control by the A allele of the TNFα.− 308 polymorphism. The use of anti-TNF therapy in individuals carrying the A allele may contribute to the reduction of disease symptoms. We consider that further studies are necessary to recommend this treatment in patients with specific symptoms of COVID-19, although this therapy has already been recommended in patients with acute respiratory distress syndrome (ARDS) within 2 days following hospital admission [33]. The use of this therapy has also been suggested for outpatients, as a treatment in high-risk individuals with COVID-19, such as elderly patients with comorbidities that can be appropriately monitored [34].

## Conclusions

It can be concluded that this study identified the effect of the TNFα.− 308A allele on the clinical states of SARS-CoV-2 infection in Cuban patients. It has an influence on developing symptoms of COVID-19, and in the case of the eastern region of the country, the risk of presenting severe forms. These findings suggest a novel marker that can be explored to identify new criteria for COVID-19 patient stratification in the country. It could be used to select personalized treatments for these patients following population-based prevention strategies.

## Data Availability

The datasets used and/or analyzed during the current study are available from the corresponding author on request.
